# Supplementary material for: Tuberculosis treatment outcome: The case of women in Ethiopia and China, ten-years retrospective cohort study
Source: PLoS One. 2019 Nov 18;14(11):e0219230. doi: 10.1371/journal.pone.0219230 (PMC6860933; doi:10.1371/journal.pone.0219230)
Supplement: Attachment — Submitted filename: Response to reviewer.docx [file pone.0219230.s003.docx]

5. Review Comments to the Author

Please use the space provided to explain your answers to the questions above. You may also include additional comments for the author, including concerns about dual publication, research ethics, or publication ethics. (Please upload your review as an attachment if it exceeds 20,000 characters)
Reviewer #1: Retrospective analysis of cohorts in two countries were made and data was analyzed. Following points were highlighted during the review:

Author’s appreciation

We would like to thank the reviewer for careful and thorough reading of this manuscript and for the thoughtful comments and constructive suggestions, which help to improve the quality of this manuscript. Our response follows (the reviewer’s comments are in non-italic form).

1. Significant number of tuberculosis cases and death were observed in Tigray than Zigong region. Highlighting the shortcomings in implementation of TB program in northern part of Ethiopia

**Dear reviewer**: I add reasons to explain the short comings in implementation of TB program a Tigray region in each discussion.
2. Why, No data is available for TB-HIV co-morbid condition in Zigong region.

**Dear reviewer:** There was a separate registration form for TB-HIV co-morbid which was not allowed to access. I did many attempts with my professor to have the information but it was not possible.

3. Factors responsible to unsuccessful treatment outcome in Tigray need to be highlighted at large.

**Dear reviewer:** I highlighted many factors responsible to unsuccessful treatment outcome in the discussion part also
4. Factors responsible for less treatment success in Re-treatment cases needs to be highlighted also,

**Dear reviewer:** the reason I highlighted is “The less treatment successes seen in the retreatment cases in Tigray could be the presence of high number of multidrug resistance TB in retreatment cases and with late introduction of drug sensitivity test in the region made the cases to take anti TB drug without knowing there MDR status.

5. In Line 75,76 stating the lacunae of the study outcomes; Thus, globally this time we lack specific proof which shows level of treatment outcomes and its factor affecting in these vulnerable group:

Dear reviewer: Many of the study compare men treatment outcome with women. But, there are very few studies which try to compare the treatment outcome among women. Hence, based on this I considered as we luck specific proof which show the level of treatment outcome

6. Why only these two regions of these two countries are chosen for comparison, as the population, and other demographic conditions of these two countries are totally different. Reason needs to be provided. However, such study needs to be planned to highlight the lacunae in implementation of the national TB control programmer and to study successful treatment outcomes.

**Dear reviewer:** The main reason to chooses this regions were ,Tigray region which is located in northern part of Ethiopia has best performance in TB prevention and treatment program and Zigong has the least performance in tb prevention and treatment and little rural among Sichuan provinces. Therefore, select these regions will show as comparable level of treatment outcome of the country.

Reviewer #2: 1. Whether the study population was native to the said region or migrant population also there needs to be clarified

**Dear reviewer:** This study did not consider the cases weather it is migrant or non-migrant because this information was not found in the TB registration logbook of Tigray region where as in Zigong there is but the number of migrant cases is not as significant.

2. since the occupation of people from Tigray is mentioned. Same needs to be highlighted about the study subjects from Zigong. Also of any correlation is deduced from the same.

**Dear reviewer:** I did not mention any occupation in Tigray
3. Any positive family history of TB in study subjects should also been recorded.

**Dear reviewer:** Yes, it was good to record any positive family history. But, both registrations did not indicate any positive family history. Besides, there is column which indicates number of contacts person which used to trace contact weather they are infected or not.

4. It is mentioned that retreatment or MDR cases showed poor treatment success as compared to those with HIV confection needs to be elaborated upon.

**Dear reviewer:** Here is the sentence on line 257-259 which says , HIV co infected TB cases were 1.59 times more likely to have treatment success (95% CI: 2.47, 5.18) compared with Unknown HIV status. In the result part and the reason behind was while The TB/ HIV prevention and treatment guideline indicates that any TB case should be tested for HIV and linked to Pre-ART treatment. But, some TB patent reject the HIV testing offer and delay themselves to take correct treatment protocol as well some health facility forgot to offer the test and delay in linking to ART program this made them to show poor treatment outcome than those known HIV positives

6. PLOS authors have the option to publish the peer review history of their article ([what does this mean?](https://journals.plos.org/plosone/s/editorial-and-peer-review-process#loc-peer-review-history)). If published, this will include your full peer review and any attached files.
If you choose “no”, your identity will remain anonymous but your review may still be made public.
**Do you want your identity to be public for this peer review?** For information about this choice, including consent withdrawal, please see our [Privacy Policy](https://www.plos.org/privacy-policy).

Reviewer #1: No

Reviewer #2: No

[NOTE: If reviewer comments were submitted as an attachment file, they will be attached to this email and accessible via the submission site. Please log into your account, locate the manuscript record, and check for the action link "View Attachments". If this link does not appear, there are no attachment files to be viewed.]
